# Items

**Published:** 1898-06

**Authors:** 


### ITEMS.

WHETHER or not the military and naval forces of the government at present under arms are to see much active service or participate in many pitched battles, it is probable that many minor casualties will occur and that the surgeons will be called upon to treat a considerable number of minor wounds, such as burns, abrasions, small cuts, and the like, for which it would seem as if the new local anesthetic, orthoform, should prove of peculiar service. It will be remembered that orthoform is a fine white powder, claimed to be entirely nonpoisonous and to possess the power of producing complete and continuous anesthesia when applied to denuded surfaces or whenever it can be brought in direct contact with sensitive nerve-ends. As the drug is also a distinct antiseptic it needs no sterilisation. It can be applied pure as a powder, or in 10 to 20 per cent. ointment with any desired base. Messrs. Victor Koechl & Co., 122 Hudson street, New York, the American agents, offer to send literature and samples to any medical officer requesting the same.

---

THE Mellier Drug Company, of St. Louis, has issued an engraving of the first meeting of the Medical society of London, which was held in 1773. The group is composed of the following-named: John Coakley Lettsom, president; William Saunders, Relph, Sir John McNamara Hayes, William Woodville, Edward Bancroft, James Ware, Thomas Bradley, James Sims, Edward Jenner, Robert Hooper, Edward Ford, Robert Blair, William Babington, John Haighton, Robert John Thornton, John Shadwell and John Aikin. This is the most interesting picture that has of late been sent out to the profession. A copy will be mailed to any physician applying for it by the proprietors of the tongaline preparations, the Mellier Drug Company, No. 2112 Locust street, St. Louis.

---

THE American agents, Messrs. Victor Koechl & Co., announce a reduction in the price of Dr. Knorr's antipyrin. In lots of 100 ounces or more it is offered at 70 cents an ounce; in lots of less than 100 ounces, at 80 cents an ounce.

---

MEDICAL excursion in June—(Denver to Salt Lake City)—The medical excursion in June will leave Denver for Salt Lake City—the Zion of the new world—on the last day of the meeting of the American medical association and the two successive days via the Rio Grande

Western railway in connection with the D. & R. G. and Colo. Mid. lines. The rate will be but $18 for the round trip, offering a trip of 1,500 miles through the Rocky and Wasatch mountains. No European trip of equal length can compare with it in grandeur or wealth of novel interest. Salt Lake City and vicinity is one grand sanitarium. The Great Salt Lake or Dead Sea of America with its magnificent bathing resort, the hot and warm springs, drives, parks, canyons and reserves are all located in or about the city. Send two cents to F. A. Wadleigh, Salt Lake City, for copy of pamphlet.

---

THE American medical association announces railroad rates for the Denver meeting, June 7-10, 1898, as follows: The Western passenger association has granted a rate to Denver and return of one-half fare, plus $2.00, thirty-day limit, for business from Chicago, St. Louis and intermediate points. Tickets on sale June 2d, 4th and 5th east of the Missouri river; 5th and 6th west of the Missouri river. A round-trip rate of $20.00, thirty-day limit, from Ogden and Salt Lake, is also announced. Application for similar rates has been made to all other passenger associations and to railroads not controlled by them. Announcement of other rates and rules governing the sale of tickets will be made in the *Journal* of the Association as soon as decisions are received. This rate is as low as granted any convention this year. J. W. Graham, M. D., chairman committee of arrangements.

---

FOR the meeting of the American medical association, to be held at Denver, Col., in June, 1898, we take pleasure in announcing that the Missouri Pacific railway has arranged to run a special through train from St. Louis to Denver, to be known as the "Chutmuck Special," making the trip via Kansas City, Pueblo and Colorado Springs.

This will be one of the handsomest trains ever run in the west, consisting of compartment sleeping cars, dining car, buffet car, etc., affording special accommodations for the wives and families of yourself and friends. Physicians should remember this in making their arrangements. Address H. C. Townsend, general passenger and ticket agent, or B. H. Payne, assistant general passenger and ticket agent, St. Louis, Mo.

